# Decreased root hydraulic traits in German winter wheat cultivars over 100 years of breeding

**DOI:** 10.1093/plphys/kiaf166

**Published:** 2025-04-24

**Authors:** Juan C Baca Cabrera, Jan Vanderborght, Yann Boursiac, Dominik Behrend, Thomas Gaiser, Thuy Huu Nguyen, Guillaume Lobet

**Affiliations:** Institute of Bio- and Geoscience, Agrosphere (IBG-3), Forschungszentrum Jülich GmbH, Wilhelm-Johnen-Str., Jülich 52428, Germany; Institute of Bio- and Geoscience, Agrosphere (IBG-3), Forschungszentrum Jülich GmbH, Wilhelm-Johnen-Str., Jülich 52428, Germany; Institute for Plant Sciences of Montpellier (IPSiM), Univ Montpellier, CNRS, INRAE, Institut Agro, Montpellier 34060, France; Institute of Crop Science and Resources Conservation, University of Bonn, Katzenburgweg 5, Bonn 53115, Germany; Institute of Crop Science and Resources Conservation, University of Bonn, Katzenburgweg 5, Bonn 53115, Germany; Institute of Crop Science and Resources Conservation, University of Bonn, Katzenburgweg 5, Bonn 53115, Germany; Institute of Bio- and Geoscience, Agrosphere (IBG-3), Forschungszentrum Jülich GmbH, Wilhelm-Johnen-Str., Jülich 52428, Germany; Earth and Life Institute, UC-Louvain, Croix du sud, 1348 Louvain-la-Neuve, Belgium

## Abstract

Wheat (*Triticum aestivum* L.) plays a vital role in global food security, and understanding its root traits is essential for improving water uptake under varying environmental conditions. This study investigated how over a century of breeding has influenced root morphological and hydraulic properties in 6 German winter wheat cultivars released between 1895 and 2002. Field and hydroponic experiments were used to measure root diameter, root number, branching density, and whole root system hydraulic conductance (*K*_rs_). The results showed a significant decline in root axes number and *K*_rs_ with release year, while root diameter remained stable across cultivars. Additionally, dynamic functional-structural modeling using the whole-plant model CPlantBox was employed to simulate *K*_rs_ development with root system growth, revealing that older cultivars consistently had higher hydraulic conductance than modern ones. The combined approach of field phenotyping and modeling provided a comprehensive view of the changes in root traits arising from breeding. These findings suggest that breeding may have unintentionally favored cultivars with smaller root systems and more conservative water uptake strategies under the high-input, high-density conditions of modern agriculture. The results of this study may inform future breeding efforts aimed at optimizing wheat root systems, helping to develop cultivars with water uptake strategies better tailored to locally changing environmental conditions.

## Teaser

Breeding over the past century reduced root axis number and hydraulic conductance in German winter wheat, potentially favoring more conservative water uptake strategies.

## Introduction

Wheat (*Triticum aestivum* L.) is one of the world's most important staple crops, occupying the largest share of cultivated land and supplying approximately one-fifth of food calories and proteins globally (Erenstein et al. 2022). Wheat yields increased considerably during the 20th century as a result of breeding programs and modern agricultural management practices, but a tendency towards yield stagnation has been observed across Europe in recent decades (Le Gouis et al. 2020). With global demand expected to increase by 50% by 2050 (FAO 2017), the pressure on agricultural systems to support this demand will intensify. This challenge will be further exacerbated by the effects of climate change, particularly rising global temperatures and changing rainfall patterns, which threaten to destabilize wheat production across regions (Challinor et al. 2014). Understanding the evolution of root traits through breeding could provide valuable insights into potential avenues for yield improvement, as roots play a central role in water and nutrient uptake.

It has been suggested that targeting root traits in breeding could lead to significant gains in wheat productivity and potentially herald a “second Green Revolution” (Lynch 2007). In particular, root architecture and root hydraulic traits are crucial to crop functioning and productivity (Torres-Ruiz et al. 2024). Historically, however, wheat breeding programs have focused primarily on selecting for yield and aboveground traits, often overlooking root traits (Waines and Ehdaie 2007). This is partly due to the technical difficulties of root phenotyping, which is more challenging than analyzing aboveground organs (Atkinson et al. 2019), as well as the complex plasticity of root traits in response to environmental cues (Schneider and Lynch 2018). Despite this, recent studies have shown that plant breeding has inadvertently affected wheat root system architecture traits such as root system size, number of roots, or root angles (Fradgley et al. 2020; McGrail and McNear 2021). However, a detailed analysis of the effects of breeding on root diameter—a plastic trait that responds to factors such as water and nutrient availability, soil structure, or temperature (Hodge 2010; Rich and Watt 2013) and plays a key role in root water uptake (Awad et al. 2018; Heymans 2022)—is still lacking, particularly regarding differences between root types. Even less is known about how breeding has influenced whole root system conductance (*K*_rs_), a key plant trait that determines the capacity of the root system to take up water at a specific evaporative demand. *K*_rs_ integrates the root system architecture and radial and axial water flows within the root system (Baca Cabrera et al. 2024), providing insights into potential adaptations in root water uptake under changing environmental conditions. While an increase in *K*_rs_ during the domestication process from wild to modern cultivated wheat has been reported (Zhao et al. 2005), it remains unclear whether—and to what extent—*K*_rs_ differs between old and modern wheat cultivars.

One possible reason why the effect of breeding on *K*_rs_ has not been (at least to our knowledge) investigated thus far is the inherent technical challenges associated with its measurement. While phenotyping methods for root architecture traits are well established for field experiments and are relatively straightforward (York 2018), the most common *K*_rs_ measurement methods are laboratory-based and labor-intensive (Boursiac et al. 2022b). Alternatively, mechanistic root water uptake modeling offers a promising approach to bridge these complementary methods, facilitating the identification of root hydraulic phenotypes across crop species and growth environments (Cai et al. 2022). Crucially, functional-structural modeling also allows for a detailed analysis of plant growth and *K*_rs_ development (Baca Cabrera et al. 2024), shedding light on their potential interactions and how these dynamics may be influenced by breeding. Such an approach, combining field and lab measurements with dynamic modeling, would provide a more comprehensive understanding of how breeding may have impacted root structure and function over time.

In this context, this work focused on the effect of breeding on the morphological traits of seminal, crown, and lateral roots, as well as the hydraulic conductance of whole root systems in wheat. We differentiated root traits of crown, seminal, and lateral roots, as they are morphologically and functionally different (Gregory et al. 1978; Nakhforoosh et al. 2014). For this, 6 German winter wheat varieties were selected, released between 1895 and 2002 and which were previously grown in the long-term experiment Dikopshof, in Germany (Schellberg and Hüging 1997). With approximately 20-yr intervals between the release of each variety, varieties were selected under increasing fertilizer input and nutrient availability (Ahrends et al. 2018; Rueda-Ayala et al. 2018). These conditions may have favored cultivars that are less competitive as individuals, making them better suited to high-input, high-density agricultural systems (Fradgley et al. 2020). Additionally, greater nutrient availability may have reduced the need for large root systems, allowing more assimilates to be directed toward increasing yield. This development may have also indirectly reduced the root water uptake capacity of wheat cultivars. Whether—and to what extent—this has been the case, remains poorly understood.

We hypothesize that breeding for yield in high-input agricultural environments may have inadvertently altered root hydraulic properties of wheat, favoring plants with low root system hydraulic conductance. To address this hypothesis, we investigated the changes in root traits across 6 German cultivars released over a period of more than 100 years, representing a breeding history gradient. Specifically, we analyzed the effect of cultivar release year (i.e. the year the cultivars were introduced for commercial use) on (i) root diameter, root axis number, and lateral branching density of wheat plants grown in the field, and (ii) whole root system conductance (*K*_rs_) and its interactions with root system development. For this, we used a pipeline integrating field-based phenotyping with detailed laboratory measurements and state-of-the-art whole-plant modeling (CPlantBox, Giraud et al. 2023). This pipeline provided a comprehensive view of the development of *K*_rs_ across different wheat varieties, highlighting how over a century of breeding may have affected root hydraulic properties and led to shifts in root water uptake strategies. These findings have important implications for future water use and drought resilience in wheat agriculture, especially in the context of a changing climate.

## Results

### Root diameter and root axes number variation with breeding

In order to investigate the changes in root traits of wheat with breeding, we conducted a field experiment over 2 growing seasons (2022 to 2023 and 2023 to 2024) under conventional agricultural management practices, using 6 German winter wheat cultivars. The cultivars span over 100 years of breeding, based on their release year: (i) S. Dickkopf—1895, (ii) SG v. Stocken—1920, (iii) Heines II—1940, (iv) Jubilar—1961, (v) Okapi—1978, and (vi) Tommi—2002. These cultivars have been used in previous studies on breeding effects in wheat (Ahrends et al. 2018; Rueda-Ayala et al. 2018; Hernández-Ochoa et al. 2023; Rezaei et al. 2024) and were selected based on their historical prominence, seed availability, and inclusion in the long-term field experiment Dikopshof (Schellberg and Hüging 1997) (see Materials and Methods section). At the end of the tillering phase, root samples were obtained with the “shovelomics” method (York 2018) and analyzed to determine the changes in root diameter, root axes number, and lateral branching density with cultivar release year. The sampling was performed simultaneously for all cultivars, in 1-d campaigns at the end of the tillering phase in both growing seasons (sampling during spring 2023 and spring 2024, respectively).

**Figure 1.**
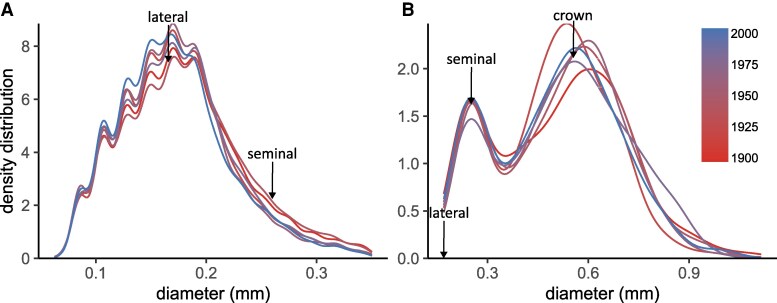
Root diameter density distribution for 6 different cultivars of winter wheat (*T. aestivum* L.). Data corresponds to lateral **A)** and axile **B)** roots obtained from the field with the shovelomics technique, for 2 experimental years (*n* = 27 to 32 plants). Density plots of lateral and axile roots were separated for visualization purposes. The arrows are a reference of the median value, for the different root types. The color scale indicates the cultivar release year.

**Figure 2.**
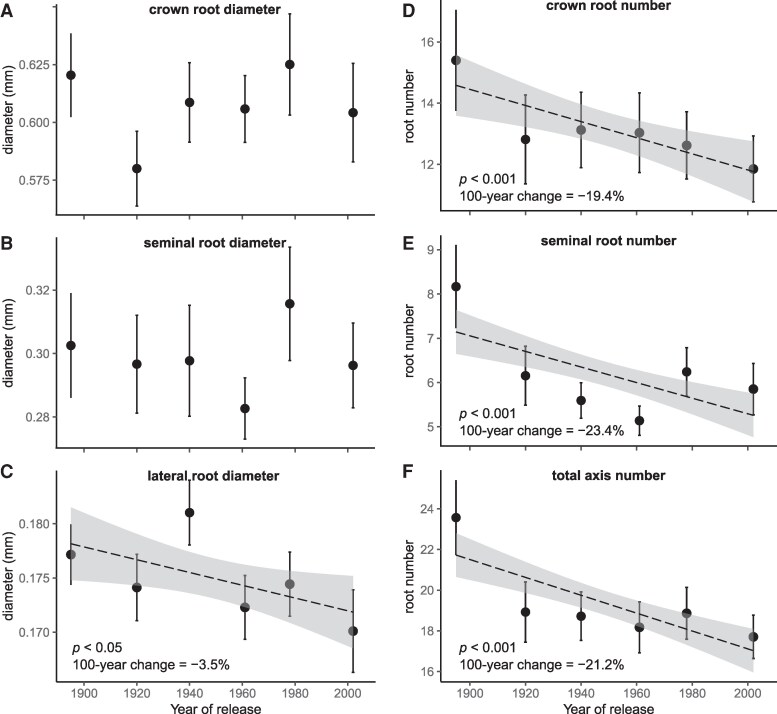
The relationship between year of cultivar release and root morphological traits for 6 different cultivars of winter wheat (*T. aestivum* L.). Panels **A** to **C)** show root diameter, and panels **D** to **F)** show number of root axes for different root types. Data points and error bars represent the mean ± CI95% across 2 years of field experiment (*n* = 27 to 32 plants). The dashed lines and the shaded areas represent the regression line ± CI95% (only shown if significant, *P* < 0.05). Statistical significance was assessed using linear mixed models. Data were log-transformed prior to model fitting and back-transformed for visualization.

Root diameter distributions of crown, seminal, and lateral roots reflected systematic differences among root types (Fig. 1, *P* < 0.001), with narrow variation in mean diameter among cultivars: crown roots = 0.58 to 0.63 mm, seminal roots = 0.28 to 0.32 mm and lateral roots = 0.17 to 0.18 mm (Fig. 2, A to C; Table 1). Accordingly, there was no significant effect of breeding (based on cultivar release year) on crown and seminal root diameters (Fig. 2, A and B; Table 2). For lateral roots, a significant decrease in root diameter with release year was observed (Fig. 2C; *P* < 0.05). However, the decrease was very small, with an average decrease per 100 years of 3.5% (Table 2). This corresponds to a decrease of 0.000063 mm yr^−1^, taking the oldest cultivar as the reference.

**Table 1.** Root morphological and hydraulic traits for 6 different cultivars of winter wheat (*T. aestivum* L.)

		Cultivar name (year of release)					
	Parameter	S. Dickkopf (1895)	SG v. Stocken (1920)	Heines IV (1940)	Jubilar (1961)	Okapi (1978)	Tommi (2002)
(a)	crown root diameter (mm)	0.62 ± 0.009	0.58 ± 0.008	0.61 ± 0.009	0.61 ± 0.007	0.63 ± 0.011	0.6 ± 0.01
	seminal root diameter (mm)	0.30 ± 0.008	0.30 ± 0.008	0.30 ± 0.009	0.28 ± 0.005	0.32 ± 0.009	0.30 ± 0.007
	lateral root diameter (mm)	0.18 ± 0.001	0.17 ± 0.002	0.18 ± 0.002	0.17 ± 0.001	0.17 ± 0.002	0.17 ± 0.002
	crown root number	15.4 ± 0.8	12.8 ± 0.7	13.1 ± 0.6	13.0 ± 0.7	12.6 ± 0.6	11.9 ± 0.5
	seminal root number	8.2 ± 0.5	6.2 ± 0.3	5.6 ± 0.2	5.1 ± 0.2	6.2 ± 0.3	5.9 ± 0.3
	tiller number	6.6 ± 0.2	4.8 ± 0.1	4.2 ± 0.1	5.6 ± 0.2	4.9 ± 0.2	4.1 ± 0.2
	branching density (cm^−1^)	1.1 ± 0.05	1.16 ± 0.15	1.1 ± 0.05	0.98 ± 0.06	0.91 ± 0.04	1.09 ± 0.06
(b)	root surface area (cm^2^)	7.8 ± 0.5	7.6 ± 0.9	7.4 ± 0.5	7.4 ± 0.9	7.8 ± 0.6	6.4 ± 0.6
	total root length (cm)	71.5 ± 4.7	69.2 ± 9.1	69.5 ± 6.2	66.4 ± 8.3	70.4 ± 6.0	57.9 ± 6.1
	*K_r_*_s_ (m^3^ MPa^−1^ s^−1^ × 10^−10^)	1.3 ± 0.2	1.1 ± 0.2	1.3 ± 0.2	1.2 ± 0.2	0.9 ± 0.1	0.7 ± 0.1
	*K_r_*_s_area_ (m MPa^−1^ s^−1^ × 10^−7^)	1.7 ± 0.2	1.5 ± 0.2	1.7 ± 0.2	1.6 ± 0.1	1.2 ± 0.1	1.1 ± 0.1
	*K_r_*_s_length_ (m^3^ MPa^−1^ s^−1^ m^−1^ × 10^−10^)	1.9 ± 0.2	1.6 ± 0.1	1.8 ± 0.3	1.8 ± 0.1	1.3 ± 0.2	1.2 ± 0.1

Plants were grown in the field in dense canopies (a) or as individual plants in hydroponic medium in the laboratory (b). Root traits of field-grown plants were determined during the tillering phase using shovelomics (*n* = 27 to 32). Root hydraulic traits were measured with a pressure chamber in 10- to 12-d-old plants (*n* = 8 to 12) in the lab. Values correspond to the mean ± SE.

**Table 2.** Effect significance (*P*-value) and regression slope of the relationship between year of cultivar release and root morphological and hydraulic traits of winter wheat (*T. aestivum* L.)

	Effect of year of release		
Parameter	*P*-value	% Change per 100 years	*n*
crown root diameter (mm)	0.74	+0.6%	27 to 32
seminal root diameter (mm)	0.92	+0.3%	26 to 32
lateral root diameter (mm)	**< 0.05**	−3.5%	26 to 32
crown root number	**< 0.001**	−19.4%	27 to 32
seminal root number	**< 0.001**	−23.4%	26 to 32
tiller number	**< 0.001**	−31.3%	26 to 32
branching density (cm^−1^)	0.27	−9.2%	26 to 32
root surface area (cm^2^)	0.22	−13.7%	8 to 12
total root length (cm)	0.19	−15.1%	8 to 12
*K_r_*_s_ (m^3^ MPa^−1^ s^−1^ × 10^−10^)	**< 0.01**	−51.8%	8 to 12
*K_r_*_s_area_ (m MPa^−1^ s^−1^ × 10^−7^)	**< 0.01**	−38.1%	8 to 12
*K_r_*_s_length_ (m^3^ MPa^−1^ s^−1^ m^−1^ × 10^−10^)	**< 0.01**	−36.8%	8 to 12

Significant effects are given in bold type. Statistical tests were performed on the log-transformed data. The % change in 100 years was calculated based on the back-transformed regression slope.

On the contrary, a highly significant decrease in root axes number with release year was observed for crown, seminal, and total axile roots (Fig. 2, D to F and Table 2, *P* < 0.001). Crown root number decreased on average from 15.4 to 11.9 (22.7%) and seminal root number from 8.2 to 5.9 (28.0%) between the oldest (S. Dickkopf—1895) and the most modern cultivar (Tommi—2002). Additionally, there was a highly significant linear relationship between crown root number and tiller number across cultivars (Supplementary Fig. S1, *P* < 0.001), as tiller number also decreased highly significantly with release year (Table 2, *P* < 0.001). This was not the case for the branching density of lateral roots, which was constant across all cultivars (Tables 1 and 2) and had an overall average value of 1.1 lateral roots cm^−1^.

### Whole root system conductance variation with breeding

**Figure 3.**
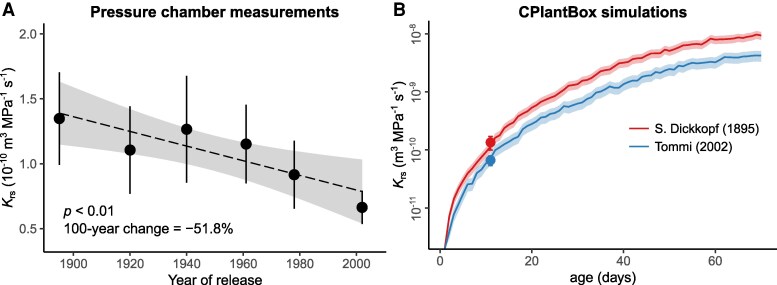
The relationship between year of cultivar release and whole root system conductance (*K*_rs_) of winter wheat (*T. aestivum* L.). **A)** shows pressure chamber measurements of *K*_rs_ in 10- to 12-d-old plants for 6 different cultivars, with data points and error bars representing the mean ± CI95% (*n* = 8 to 12 plants). The dashed line and shaded area represent the regression line ± 95%. Statistical significance was assessed using ordinary least squares regression. **B)** shows the development of *K*_rs_ over plant age for the winter wheat cultivars S. Dickkopf (release year 1895) and Tommi (release year 2002). The line and the shaded area correspond to the mean ± SE of *K*_rs_ simulations (*n* = 6 simulation runs) using the whole-plant model CPlantBox. Data in **(A)** were log-transformed prior to model fitting and back-transformed for visualization. Notice the log-scale in panel **(B)**.

The same cultivars used in the field experiment were grown in a hydroponic medium in the laboratory to measure the hydraulic conductance of whole root systems (*K*_rs_) in young plants (10 to 12 days old, with no crown roots), using the pressure chamber technique. *K*_rs_ showed a range of variation from 1.3 × 10^−10^ (the oldest cultivar) to 0.7 × 10^−10^ m^3^ MPa^−1^ s^−1^ (the most modern cultivar), which corresponded to a highly significant decrease with cultivar release year (Tables 1 and 2, Fig. 3A, *P* < 0.01). On average, *K*_rs_ decreased 51.8% in a 100-yr period (Table 2). A similar significant negative trend with release year was observed for *K*_rs_ normalized by root system surface area (*K*_rs_area_) or total length (*K*_rs_length_, Supplementary Fig. S2), but those trends were less pronounced (38.1% and 36.8% decrease in 100-yr period, respectively).

To complement these early-stage measurements, the development of *K*_rs_ with plant growth was modeled using the whole-plant model CPlantBox (Giraud et al. 2023), including the dynamics of tillering and crown root growth. The model was parametrized for the 2 most contrasting cultivars (oldest vs. newest) based on our measurements of root architecture and *K*_rs_ (Supplementary Table S1). For both cultivars, a nonlinear relationship between age and *K*_rs_ was observed, with a very steep increase of *K*_rs_ during the first 20 to 30 days and a flattening out of the curve until the end of the simulation (Fig. 3B). For both cultivars, *K*_rs_ increased ≈ 3 orders of magnitude throughout the simulated growing period, primarily due to the large increase in total root length (exceeding 120 m for cultivar S. Dickkopf and 63 m for cultivar Tommi, respectively; Supplementary Fig. S3). Cultivar Tommi showed a consistently lower *K*_rs_ than cultivar S. Dickkopf, which was in line with the chamber pressure measurements. *K*_rs_ of Tommi was between ca. 35% and 60% lower than that of S. Dickkopf (average difference 50.8%) and the difference between cultivars was most pronounced at the end of the simulation period (Fig. 3B). The model was also capable of capturing differences between cultivars in terms of *K*_rs_area_ and *K*_rs_length_. For both parameters, the model showed approximately 25% lower values for Tommi compared with S. Dickkopf, at the time when the pressure chamber measurements were taken (10 to 12 day-old plants).

## Discussion

### Root axes number declined, but root diameters were unaffected by breeding

This study analyzed the variation of root morphological and hydraulic traits among 6 German wheat cultivars, spanning over 100 years of breeding history based on their release year. A key finding of our research was the significant decline in root axes (crown, seminal, and total axile roots) with release year, consistent with trends in wheat cultivars from the United States (McGrail and McNear 2021), the United Kingdom and Northern Europe (Fradgley et al. 2020), and China (Zhu et al. 2019). This decline may be a result of unconscious breeding for smaller root systems, which would reduce below-ground competition, improving resource use. As selection probably occurred under high-input management (which is typical of agroecosystems, particularly in Germany), phenotypes with fewer axes may have been prioritized, which would impact root system architecture overall. We did not directly measure root angles or total root biomass; thus, we cannot precisely quantify the effect of reduced root axis numbers on root system size and root architecture at different developmental stages. However, a strong relationship between the number of roots and root system size has been reported for wheat (Maqbool et al. 2022). Moreover, CPlantBox simulations indicated that S. Dickkopf (the oldest cultivar) had an almost 2-fold greater root system length compared with Tommi (the most modern cultivar) at the end of the simulation period (Supplementary Fig. S3), primarily due to the higher number of root axes. Whether the decrease in root axes with cultivar release year also promoted changes in root system architecture, such as a shift toward the steep and deep root ideotype (Lynch 2013), should be explored in future studies.

Notably, the decrease in root axes with release year was strongly shaped by the oldest cultivar, which had significantly more axile roots than all others (*P* < 0.001, Tukey post hoc test, Supplementary Table S2), with reduced variation in root axes after 1920, pointing to homogenization among cultivars. Similar patterns were found in US cultivars classified as either old (<1935), intermediate (1970 to 1989) or modern, where large differences in root traits were observed between the old cultivars and the intermediate and modern ones, but not between the last 2 groups (McGrail and McNear 2021). Limited phenotypic diversity in modern cultivars may explain this, as they originated from just 2 ancestral wheat groups, maintaining haplotype integrity (Cheng et al. 2024). Also, as there are only a handful of genes involved in crown root formation in wheat (Xu et al. 2021), they may have been involuntarily counter-selected early in breeding programs.

Moreover, our data revealed a highly significant positive relationship between crown root number and tiller number, for all cultivars (*P* < 0.001, Supplementary Fig. S1), suggesting that crown root number variation was linked to size rather than to changes in node number per tiller. Modern cultivars typically have smaller root systems (Waines and Ehdaie 2007) and less tillers (Fang et al. 2011) than older ones, which possibly indicates adaptation to high-density planting. Zhu et al. (2019) found that newer cultivars produce higher yields only at higher sowing densities, suggesting changes in competitive behavior. Our findings point in that direction, as modern cultivars with fewer root axes are better suited for reducing intra-crop competition and maximizing yield.

On the contrary, root diameters showed negligible variation among cultivars. Distinct average diameters were observed for crown (0.58 to 0.63 mm), seminal (0.28 to 0.32 mm), and lateral roots (0.17 to 0.18 mm), with significant differences among root types (*P* < 0.001), which was consistent with previous studies showing systematically bigger diameters in crown roots than in seminal roots across wheat accessions (Xu et al. 2021). However, there were no significant trends in root diameters with release year, except for a significant decrease in lateral root diameter (3.5% per 100 years). In terms of water uptake capacity, a decrease in lateral root diameter of 0.1 mm (i.e. the difference between the oldest and the newest cultivar) would result in less than a 0.1% decrease in *K*_rs_ or root system volume, according to CPlantBox simulations. However, the observed changes in lateral root diameter—albeit small—might underlie adaptations in resource acquisition strategies with breeding, such as suitability for mycorrhizal colonization, as has been shown in maize (*Zea mays* L.) (Wild et al. 2024).

Our results indicated high stability among cultivars in root diameters, suggesting homogenization of this trait with modern breeding. Similarly, a study with 196 wheat accessions (Xu et al. 2021) found that the coefficient of variation of root diameter was the lowest among multiple root traits, both for seminal and crown roots. Likewise, Peng et al. (2019) observed no variation in average root diameter among 10 US varieties but noted effects of field site and irrigation. In contrast, Awad et al. (2018) reported significant differences in average root diameter among cultivar lines from Colorado. Interestingly, though, this experiment was conducted under drought stress only (no well-watered treatment). In our study, performed under nonstress conditions (common nutrient application and crop protection practices and precipitation above the long-term average in both growing seasons, Materials & Methods), root diameters remained stable across cultivars representing >100 yr of breeding in Germany. Whether this stability persists under stress conditions requires further investigation, especially as it has been suggested that breeding has not reduced root trait plasticity to environmental pressures in wheat (Nimmo et al. 2023). This underscores the importance of studying the interactions between breeding and environmental stress, as growth conditions strongly influence root development and morphology.

### Whole root system conductance decreased with breeding

Our study revealed a significant decrease in the conductance of whole root systems (*K*_rs_) with breeding over the past century. This trend was observed both in absolute terms (51.8% decrease over 100 years) and when *K*_rs_ was normalized by the root system surface area (*K*_rs_area_) or total root length (*K*_rs_length_), though the normalized values exhibited a less pronounced decline (38.1% and 36.8% decrease over 100 years, respectively). It is important to note that the pressure chamber measurements were performed on young plants (10 to 12 d) consisting of seminal and first-order lateral roots only (no crown roots). To complement these early-stage measurements, we utilized the CPlantBox model to simulate the changes in root system architecture and *K*_rs_ with plant development, including the dynamics of tillering and crown root growth, for the 2 most contrasting cultivars (i.e. oldest and most modern ones). Consistent with the pressure chamber measurements, the model showed that *K*_rs_ in the most modern cultivar (Tommi) was 37.5% lower compared with the oldest cultivar (S. Dickkopf) at plant age 10 to 12 d. This pattern also applied to the modeled *K*_rs_area_ and *K*_rs_length_, with both showing approximately 24% lower values in Tommi than in S. Dickkopf at that age. Additionally, the modeled *K*_rs_ remained systematically higher in S. Dickkopf than in Tommi throughout the entire simulation period, with the differences becoming more pronounced in the later stages (Fig. 3B). Moreover, the model indicated a nonlinear increase in *K*_rs_ with age for both cultivars, with a very steep rise during the first 20 to 30 days, followed by a flattening out. This nonlinear pattern has been reported for various crops and is associated with the counteracting effects of root growth, which adds more conductances to the hydraulic network—thus increasing the total conductance—and the increment in the proportion of less conductive root segments with age, causing hydraulic limitations at later stages of development (Baca Cabrera et al. 2024).

**Figure 4.**
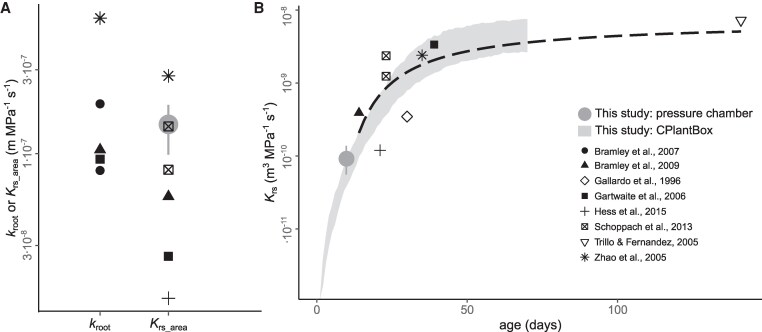
Comparison between root hydraulic properties of wheat (*T. aestivum* L.) obtained from the literature and this study. **A)** Area-normalized conductance of individual roots (*k*_root_) or whole root systems (*K*_rs_area_); and **B)** whole root system conductance (*K*_rs_) development with age. Black symbols represent literature values measured using a hydrostatic driving force under nonstress conditions (data extracted from a root hydraulic properties database, Baca Cabrera et al. 2024, *n* = 8 studies). The gray filled circles in **(A)** and **(B)** represent the mean and range of variation of pressure chamber measurements in this study (*n* = 6 cultivars). The gray shadowed area in **(B)** represents the mean ± SE of *K*_rs_ simulations using CPlantBox, as presented in Fig. 3 (*n* = 6 simulation runs). The dashed black line in **(B)** represents a fitted exponential model for the literature data (Baca Cabrera et al. 2024).

This study has investigated the effect of breeding on *K*_rs_. To better contextualize the extent of the observed decrease in *K*_rs_ and *K*_rs_area_, we compared our results with published data on wheat, for non-stressed conditions. The decrease in *K*_rs_area_ with breeding (from 1.7×10^−7^ to 1.1×10^−7^ m MPa^−1^ s^−1^) fell well within the range of variation reported in the literature, which was of more than 1 order of magnitude (1.5×10^−8^ to 5.9×10^−7^ m MPa^−1^ s^−1^, Fig. 4A). As *K*_rs_area_ already accounts for possible differences in root system size, the large range of variation in the literature must have been associated with contrasting experimental designs. This signalizes that the breeding effect on *K*_rs_area_ detected in our study could potentially be even higher in less controlled environments than the one we used (hydroponics, with no nutrient limitation). Interestingly, our measurements were on the higher end of reported values and were only clearly lower than 1 study involving plants grown in hydroponics (Zhao et al. 2005). In most of the remaining studies, the plants were grown in soil, so that the large range of variation could reflect differences in the growth medium, as has been pointed out previously (Garthwaite et al. 2006). Moreover, the *K*_rs_ development with age observed in our model aligned closely with a fitted curve based on literature data (Fig. 4B), underscoring that our findings were within a reasonable range for wheat, not only for point measurements, but likewise regarding the dynamics of *K*_rs_ and root system development.

### *K*_rs_ and root axes number decrease with breeding suggest unconscious selection for more conservative root water uptake

The present work showed that root diameter classes have remained constant, but there was a significant decrease in root axes number and *K*_rs_ with breeding, based on cultivar release year. As the cultivars used in this study have been bred under the high-input, high-density agricultural systems typical of Germany, this trend was likely related to unconscious selection for less selfish phenotypes, as has been proposed elsewhere (Waines and Ehdaie 2007; Aziz et al. 2017; Fradgley et al. 2020). This suggests that breeding has favored smaller root systems because, with high inputs of fertilizers, large root systems were less necessary for efficient nutrient uptake. Notably, it was shown for Australian wheat cultivars that selection for yield reduced total root length, while increasing nitrogen uptake per unit root length, indicating a trend toward smaller, more efficient root systems with breeding (Aziz et al. 2017). Such a reduction in root system size would also lead to a decrease in *K*_rs_ in modern cultivars. Consequently, breeding for yield may have indirectly favored genotypes with more conservative characteristics regarding their root water uptake capacity.

It is noteworthy that the most pronounced differences in root axes number were observed between the oldest cultivar and the more recent ones, suggesting a potential homogenization and limited phenotypic diversity in modern wheat cultivars (McGrail and McNear 2021; Cheng et al. 2024). To further investigate how breeding has influenced root hydraulic traits, future studies could focus on a larger panel of cultivars released after the Green Revolution, with particular emphasis on *K*_rs_ measurements. Such targeted studies may provide additional insights into breeding-driven variation in root water uptake capacity and could inform future breeding efforts.

In rainfed agricultural systems, like the one where our experiment was conducted and which is common for wheat cultivation in Germany, low *K*_rs_ could be advantageous, especially with drought events becoming more common in the future. Plants with low root hydraulic conductance can potentially conserve water during early growth, allowing for more efficient use at later developmental stages (Passioura 1972). In fact, low axial conductance has been identified as a key trait for supporting sustainable grain yield under drought conditions in wheat. However, the advantage of low *K*_rs_ in terms of water use efficiency also depends on aboveground canopy development—a factor we did not analyze here— as the water demand imposed by large leaf area could revert water savings. In this regard, the relationship between root system conductance, water use, canopy development, and yield with breeding should be addressed in more detail in future studies, particularly under drought conditions.

As previously mentioned, this study was performed under nonstress conditions. Nevertheless, the observed decrease in *K*_rs_area_ per 100 years of breeding history (38.1%) resembled the difference between an elite drought-tolerant and a drought-sensitive cultivar from Australia under well-watered conditions (ca. 40% difference, Schoppach et al. 2013). However, interpreting changes in water uptake strategies based on this comparison is challenging, due to the stark differences between the drought-prone climate of Australia and Germany's rather moist and mild climate, as well as the differing breeding pressures. Additionally, the direct impact of decreased *K*_rs_ on total water use remains uncertain, since transpiration was not measured alongside shovelomics and root hydraulic measurements. Interestingly, though, Schoppach et al. (2017) found in wheat cultivars released between 1890 and 2008 that breeding for yield inadvertently favored genotypes with a limited-transpiration trait, optimizing water use to meet evaporative demand and enhancing water conservation. In addition, measurements from our field experiment after flowering indicated a trend of reduced canopy transpiration and per tiller transpiration in the more modern cultivars (Supplementary Fig. S4). While these findings suggest unconscious selection for root traits associated with more conservative root water uptake, the observed decrease in *K*_rs_ may also reflect an unintended side effect of root system adaptations to the high-input agricultural systems typical of Germany, such as the decrease in root axis number, which reduces inter-plant competition. Further studies combining plant transpiration, *K*_rs_, and root architecture measurements are needed to disentangle these effects.

Finally, a limitation of our study is that all measurements of *K*_rs_ were performed on plants grown in hydroponics, while root morphological traits were derived from field-grown plants. Hydroponic systems provide an idealized growth environment, free from physical soil constraints such as compaction, excessively large pores, or uneven nutrient and water distribution (Passioura 2002; Poorter et al. 2016), and exclude inter-plant competition, which significantly shapes root traits in natural canopies (Poorter et al. 2016). These differences may influence root hydraulic properties, potentially complicating the extrapolation of results from hydroponics to natural agroecosystems. Moreover, studies comparing *K*_rs_ between hydroponic and soil-grown plants report inconsistent results (Hernandez and Orioli 1985; Brar et al. 1991; Yang and Grantz 1996), emphasizing the need for cautious interpretation.

To bridge the gap between approaches, in this study we modeled root growth using field-derived shovelomics data. Although the simulations did not explicitly account for soil conditions, the model was parameterized with key traits observed in the field, including seminal and crown root number, root diameter classes, and branching density (Supplementary Table S1), to capture the main dynamics of root growth under field-like conditions and its effect on *K*_rs_ development. This integrative approach using the functional-structural model CPlantBox represented an effort toward connecting laboratory and field measurements and reducing the uncertainty on the hydroponics-based *K*_rs_ observations. Nevertheless, future studies investigating the effect of breeding on *K*_rs_ should explicitly include a dynamic representation of soil conditions and their influence on root growth and water uptake dynamics. Such modeling framework would help better account for the complexities of soil-root interactions, including physical and hydraulic heterogeneity, and improve the relevance of simulation outputs for natural agroecosystems.

### Potential drivers for changes in *K*_rs_

Our results showed not only a decrease for total *K*_rs_, but also when normalized by either total root surface area or root length (*K*_rs_area_ and *K*_rs_length_). This suggests that the decline in root system conductance with breeding was not solely due to a size effect (i.e. modern cultivars having smaller root systems), but also due to a reduced water transport capacity per unit root. While our study evidenced a size effect (i.e. a decrease with cultivar release year in the total number of root axes and root surface area before tillering, Tables 1 and 2), the mechanisms behind the decrease in conductance per unit root could not be assessed. Studies in maize have shown that declines in *K*_rs_ under phosphorus deficiency were driven by reorganization of the root system rather than solely by changes in overall size (Bauer et al. 2024) and that higher root length does not necessarily lead to higher whole root system conductance under contrasting soil types and water conditions (Nguyen et al. 2024), highlighting the complex relationship between root structure and function. These findings underscore the need for more detailed measurements at the individual root or root segment scale, which, however, laid beyond the scope of this study.

Furthermore, a key finding in our study is that *K*_rs_ and *K*_rs_area_ decreased with cultivar release year, even though root diameter classes and branching density remained constant. Root diameter has been proposed as a good proxy for root conductance (Heymans 2022), but we did not see this relationship across the selected cultivars. Similarly, Schoppach et al. (2013) found significant differences in *K*_rs_area_ between 2 wheat cultivars with contrasting drought sensitivity despite no differences in root diameter. This might be related to the fact that radial conductance—often considered the more limiting component of *K*_rs_ (Frensch and Steudle 1989)—is proportional to the root cross-sectional area to water flow, but also inversely proportional to the path length from the root-soil interface to the xylem vessels (Bramley et al. 2009). While the former depends mostly on root diameter, the latter is affected by various anatomical features (e.g. formation of apoplastic barriers and aerenchyma, cortex width, stele diameter, and root cortical senescence; Schneider and Lynch 2018; Heymans et al. 2021). Similarly, axial conductance of roots can vary based on the number and diameter of xylem vessels, independent of root diameter (Schoppach et al. 2013). Additional factors, such as aquaporin (AQP) expression and axial flow limitations, also significantly impact root water transport. In wheat, conductance reductions of up to 50% in both individual roots and entire systems following AQP inhibition have been observed (Bramley et al. 2009). Moreover, as the axial conductivity of xylem vessels can become limiting with increasing length (Boursiac et al. 2022a; Bauget et al. 2023), a decrease in whole root system conductance can occur without changes in root diameter. This decrease would result from the presence of longer roots with conductive segments farther from the base, connected by greater resistance due to increased xylem length. Clearly, there is a need for complementing our findings with anatomical data for the same (or similar) cultivars to the ones we used in our experiment, to better understand the mechanisms behind the decrease in *K*_rs_ over 100 years of breeding.

### Genetic considerations and perspectives

Although investigating the genetic basis of the observed decrease in *K*_rs_ was not the primary focus of this study, identifying specific quantitative trait loci (QTLs) would be a valuable next step in advancing our findings. Such research could help link phenotypic changes with the underlying genetic mechanisms, providing deeper insights into the factors driving long-term alterations in root hydraulic properties.

In this context, it is important to acknowledge that the root traits analyzed here, though phenotypic, are ultimately governed by genetic factors. However, direct selection on root traits has not historically been a focus in wheat breeding programs. Instead, changes in these traits likely occurred indirectly, resulting from breeding efforts targeting yield performance under high-input agricultural systems. For simpler traits such as root axes number, which we found to decrease significantly with breeding, QTLs have already been identified in wheat (Guo et al. 2012; Atkinson et al. 2015). In contrast, more complex integrative traits like *K*_rs_—which reflect the combined effects of root system architecture and hydraulic properties—are controlled by many genes with small individual effects and are further modulated by environmental conditions (Voss-Fels et al. 2018). To date, no QTLs specifically associated with *K*_rs_ have been identified in wheat, likely due to this genetic complexity and the technical challenges of measuring *K*_rs_. However, QTLs linked to *K*_rs_ have been identified in Arabidopsis, providing insights into the genetic control of this trait in model species (Shahzad et al. 2016; Tang et al. 2018). Our findings provide a valuable phenotypic foundation, highlighting how key root hydraulic traits have changed over a century of breeding. They offer a basis for future studies to identify genetic markers or QTLs linked to these long-term trends in wheat.

## Conclusions and future perspectives

Our study revealed a significant decrease in whole root system hydraulic conductance (*K*_rs_) of wheat with breeding, based on cultivar release year, as a result of both a decrease in root axes number and of *K*_rs_ per unit root area (*K*_rs_area_). This suggests that breeding has indirectly selected for root systems which are less competitive as individuals, making them more suited to modern high-input agricultural systems and potentially exhibiting more conservative root water uptake. These findings underscore the value of methods that can disentangle changes in root hydraulic architecture, as demonstrated by our integration of field root sampling, pressure chamber phenotyping, and whole-plant modeling with CPlantBox.

This pipeline captured both cultivar differences at the sampled growth stage and the dynamics of *K*_rs_ and root system development effectively narrowing the gap between hydroponic-based *K*_rs_ measurements—free from soil constraints and inter-plant competition— and the challenges of extrapolating these results to field-grown plants. Expanding the model to include soil dynamics and soil-root interactions could further enhance its applicability to natural agroecosystems.

Furthermore, our work intentionally focused on German cultivars to avoid complexities arising from differing breeding strategies influenced by climate or agricultural practices. Future studies would benefit from including cultivars from diverse provenances, providing insights into how breeding and environmental pressures interact to shape *K*_rs_ and other traits. To facilitate such analyses, a comparison of very old versus modern cultivars—where the largest differences were observed in our study—would be particularly valuable.

Despite its large economic and agricultural importance, there is a surprising scarcity of studies focused on *K*_rs_ in wheat (fewer than 10; see Fig. 4, A and B), likely due to the technical complexity of *K*_rs_ measurements in tillering grass species with fibrous root systems. The phenotyping pipeline applied here could be replicated to explore how breeding has affected root hydraulic properties under different environmental stress conditions (e.g. drought, nutrient limitation, salt stress).

Additionally, while it was not the purpose of this study, investigating the genetics underlying the decrease in *K*_rs_—such as identifying specific QTLs—would be a critical next step. Such studies would bridge the gap between phenotypic observations and genetic mechanisms, providing deeper insights into the drivers of root hydraulic evolution. As some of the largest changes in our dataset were observed between the oldest cultivar and the more recent ones, future research could focus on a broader panel of post-Green Revolution cultivars to further investigate variation in *K*_rs_ and its potential for breeding applications.

Moreover, this research framework could be applied not only to wheat but also to other tillering grass species of economic importance such as barley or rice. Also, further investigation into the effects of breeding on wheat root anatomy is essential to uncover the mechanisms driving the evolution of root hydraulic traits presented here. In particular, a promising area for future exploration involves breaking down whole root system conductance into its axial and radial components, as has been done with Arabidopsis (Boursiac et al. 2022a) and maize (Bauget et al. 2023). This would provide a clearer understanding of how each component has been affected by breeding, allowing us to disentangle their contributions to the long-term decrease in *K*_rs_ of wheat cultivars. Finally, the insights from our study could serve as a foundation for future breeding efforts aimed at optimizing wheat root systems. By focusing on root hydraulic traits, breeders may be able to develop cultivars with more conservative water uptake strategies and enhanced adaptability to changing environmental conditions. Incorporating root traits into breeding programs could help meet the challenges posed by climate change, ensuring that wheat cultivars are better equipped to thrive under increasing stress and locally changing environmental conditions.

## Materials and methods

### Field experiment description

**Figure 5.**
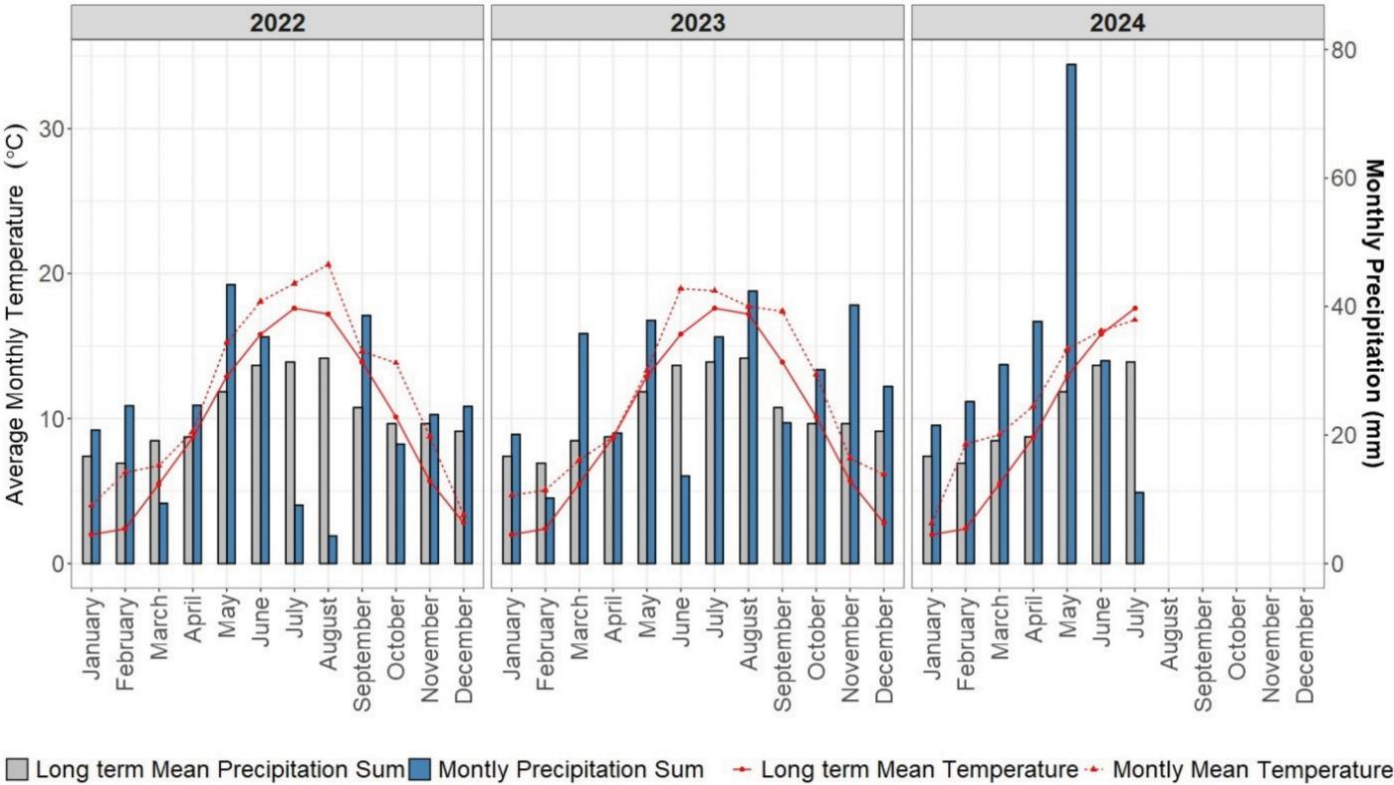
Monthly average temperature and monthly total precipitation profiles at Campus Klein-Altendorf, Germany. Lines represent monthly average temperature and bars represent monthly total precipitation measured during the years 2022, 2023 and 2024. For comparison, the long-term means (based on data from 1956 to 2014) are also presented.

A rainfed field experiment with winter wheat (*Triticum aestivum L*.) was conducted during 2 growing seasons between the years 2022 and 2024 at the research station Campus Klein-Altendorf, near Bonn, Germany (50°37′ N, 6°59′ E). Campus Klein-Altendorf is located within the temperate oceanic climate zone according to Peel et al. (2007). Long-term weather data for the years between 1956 and 2014 showed a yearly precipitation of 603 mm, a yearly mean temperature of 9.4 °C and a growing season between 165 and 170 days. Each experiment comprised an entire growing season: October 25, 2022 to July 19, 2023 and October 23, 2023 to July 31, 2024, respectively. During the experimental years, the yearly mean temperature was 11.6 °C and the yearly precipitation sum was 602.5 mm (Fig. 5). The soil is characterized as a Haplic Luvisol, developed on loess, which is known to be very homogenous (Table 3).

**Table 3.** Soil characteristics of a Haplic Luvisol from Campus Klein-Altendorf, Germany

Depth	Silt (%)	Sand (%)	Clay (%)	Texture	pH	Bulk density (g cm^−1^)	*C*_org_ (%)
0–15 cm	75.8	7.2	17.8	SiL	5.93	1.42	0.92
15–45 cm	70.3	5.1	25	SiL	6.03	1.57	0.48
45–60 cm	64.3	4.4	31.5	SiCL	5.63	1.55	0.56
60–75 cm	63.8	4.5	31.8	SiCL	5.88	1.59	0.43
75–90 cm	66.3	3.9	29.8	SiCL	6.35	1.58	0.48

Values are means of 4 soil profiles (a more detailed description can be found at Vetterlein et al. (2013)).

The experiment has been assembled as a complete randomized block design with 6 cultivars and 4 field repetitions. Six German winter wheat cultivars, spanning a range of breeding history of over 100 years, were selected. The grown cultivars, sorted by their release year were: (i) S. Dickkopf—1895, (ii) SG v. Stocken—1920, (iii) Heines II—1940, (iv) Jubilar—1961, (v) Okapi—1978, and (vi) Tommi—2002. The cultivar selection was based on historical relevance, experimental feasibility, and consistency with prior studies. Specifically, we applied the following criteria: (i) cultivars had clear records of their release dates, ensuring accurate representation of distinct breeding periods; (ii) they were prominent and widely cultivated in Germany during their release periods, according to the German Federal Plant Variety Office (Beschreibende Sortenliste 2005, ISSN 0948-4167; Beschreibende Sortenliste 1985); (iii) seed availability, particularly important for older cultivars; and (iv) inclusion in other studies investigating breeding effects on winter wheat (Ahrends et al. 2018; Rueda-Ayala et al. 2018; Hernández-Ochoa et al. 2023; Rezaei et al. 2024), enabling comparability across datasets. All selected cultivars were previously grown in the long-term fertilization experiment at Dikopshof (Schellberg and Hüging 1997). Given the technical complexity and labor-intensive nature of the root traits analyzed—particularly the measurement of whole root system hydraulic conductance—we focused on 6 cultivars from the 18 historically grown in the experiment. A systematic 20-yr interval between cultivars was applied to span the breeding history while ensuring manageable workloads and robust data.

All cultivars were sown with the target density of 320 plants m^−2^. The experimental field was managed conventionally with a mineral fertilizer application of 170 kg of N ha^−1^ and year (60 kg before sampling) and herbicide application at early growth stages before sampling. Yield features of the cultivars with regard to nutrient use efficiencies were recently investigated by Ahrends et al. (2018); Rueda-Ayala et al. (2018) and Hernández-Ochoa et al. (2023).

### Root sampling

We used a slightly modified “shovelomics” method for wheat (York 2018) to phenotype several root traits from the field experiment. The sampling was performed at the end of the tillering phase (BBCH < 30) for both growing seasons (sampling in spring 2023 and spring 2024, respectively), with all cultivars sampled simultaneously during 1-d campaigns. We excavated a representative area of the plots, with a diameter and depth of 20 to 30 cm in the topsoil. Given the high planting density, each individual sample contained around 5 to 10 plants. The sample bags with entire excavated plants were transported to the laboratory and stored at 5 °C until root washing. In the laboratory, the samples were soaked in water and then gently washed with a hose and nozzle to remove the soil, without damaging the roots. The root crowns were then severed from the shoots close to the base (with ca. 3 cm of the tillers attached) and then stored again at 5 °C in a water (37.5%)-ethanol (37.5%)-glycol (25%) solution. 3 to 4 plants per sample were preserved for further analysis.

Images of seminal and crown roots were taken for a total of 27 to 32 plants per cultivar (3 to 4 plants per plot and year). For each sample, the tillers were separated from the roots by cutting directly above the mesocotyl and then manually counted. Subsequently, seminal and crown roots were carefully separated and placed on root scanning trays filled with distilled water and scanned at 600 dpi (Epson Expression 12000XL, Epson, Japan). The scanned images were analyzed using SmartRoot (Lobet et al. 2011) to determine the following traits: number of crown and seminal roots and total axes number; crown, seminal, and lateral root diameter; and inter-branching density of lateral roots on crown and seminal roots.

### Root hydraulic conductance measurements

The same cultivars used in the field experiment were grown in the laboratory in a hydroponic medium to perform root hydraulic conductance measurements. The growing protocol has been described previously for maize plants (Bauget et al. 2023). In brief, seeds were surface sterilized with 1.5% (v/v) bleach mixed with 1 drop (≈50 *μ*L) of Tween-20 for 5 to 8 min and then treated with 35% (v/v) H_2_O_2_ for 2 min, rinsed with 70% (v/v) ethanol, and washed 6 times with sterilized water. Seeds were then germinated for 4 d in plastic boxes filled with wet clay aggregates (Agrex 3–8, Agrex Co., Portugal) and covered with a transparent plastic foil. The plastic boxes were placed in a growth chamber at 65% relative humidity, with 22 °C/20 °C and 16 h/8 h light/dark cycles (250 *μ*mol m^−2^ s^−1^ photosynthetic photon flux density). At 5 days after sowing (DAS), the plants were transferred to a hydroponic container placed in the same growth chamber and filled with hydroponic solution with the following composition:1.25 mm KNO3, 0.1 mm CaCl_2_, 1.5 mm Ca(NO_3_)_2_, 0.5 mm KH_2_PO4, 0.75 mm MgSO_4_, 0.1 mm Na_2_SiO_3_, 0.05 mm FeEDTA, 0.05 mm H_3_BO_3_, 0.012 mm MnSO_4_, 0.001 mm ZnSO_4_, 0.0007 mm CuSO_4_, 0.00024 mm Na2MoO_4_, 0.00001 mm CoCl_2_, and 1 mm MES. Air was continuously injected into the containers with a bubbling system to ensure adequate solution mixing and sufficient oxygen.

At 10 to 12 DAS root water transport was measured on de-topped plants using a set of pressure chambers, as described in Boursiac et al. (2022a) for Arabidopsis, with slight modifications. The entire root system, consisting of 3 to 6 seminal roots and their laterals, was excised directly below the seed and carefully inserted into an adapter sealed with silicon (Coltene Whaledent, France), threaded through the seal of the pressure chamber lid and placed in the pressure chamber filled with nutrient solution. The adapter was connected to a high-accuracy flowmeter (Bronkhorst, France) to record the sap flow (*J*_v_, m^3^ s^−1^) from the root system. The root system was subjected to various pressures (P, MPa) applied using nitrogen gas, and the resulting sap flow was recorded. The measurement protocol included a pre-pressurization phase of >5 min at 0.32 mPa, to achieve stability in the system, followed by measurements at pressures of 0.16, 0.24, 0,1, 0.32 and 0.24 mPa. The resulting slope of the *J*_v_(P) linear relationship was used to deduce the whole root system conductance (*K*_rs,_ m^3^ MPa^−1^ s^−1^) of the wheat cultivars. Measurements that did not show a linear *J*_v_(P) relationship were excluded from the analysis. A total of 8 to 12 measurements were obtained per cultivar.

After the measurements, the roots were placed in a tray with distilled water and scanned at 600 dpi with a desktop scanner. The images were analyzed with SmartRoot to obtain the diameter of seminal and lateral roots and the total length and surface area of the root system. To account for size effects on possible *K*_rs_ variation among cultivars, *K*_rs_ was normalized by either total root length (*K*_rs_length_, m^3^ MPa^−1^ s^−1^ m^−1^) or root surface area (*K*_rs___area_, m MPa^−1^ s^−1^). Outliers were determined using the interquartile range (1.5 × IQR) method and confirmed with the Grubbs’ test. Samples were identified as outliers when the values of *K*_rs_, *K*_rs_length_, and *K*_rs_area_ were outside the interquartile range.

### Modeling of *K*_rs_ development with age

The pressure chamber measurements delivered accurate information on *K*_rs_ at a very young plant age (10 to 12 d). However, *K*_rs_ is not a static value, as it shows a nonlinear increase with root system age (Baca Cabrera et al. 2024). Additionally, at measurement age the plants had still not developed crown roots, which are major contributors to total water uptake in grasses (Ahmed et al. 2018). To widen our analysis, we modeled the development of the root system and *K*_rs_ with age, using the 3D whole-plant model CPlantBox (Giraud et al. 2023). Simulations were performed for the oldest (S. Dickkopf) and the most modern (Tommi) cultivars for 70 d, which corresponded to plant development until the end of the tillering phase. CPlantBox simulates the development of the whole-plant architecture, which is represented as a series of segments corresponding to different plant organs (e.g. leaves, crown, and seminal roots, pseudo-stems). Plant development occurs via the elongation of previously existing segments or the creation of new ones. Water flow from the soil-root interfaces to xylem vessels at the plant collar and *K*_rs_ are dynamically simulated at each time step using the analytical solution of water flow within infinitesimal subsegments (Meunier et al. 2017), as implemented in CPlantBox (Giraud et al. 2023; Bauer et al. 2024).

For the parametrization of the whole-plant architecture in CPlantBox, we used an existing XML-input parameter file for wheat (Giraud et al. 2023) and modified it based on the root sampling data (Supplementary Table S1). Segment scale root hydraulic properties (radial conductivity *k*_r_ and axial conductance *k*_x_) needed for the simulation of *K*_rs_ were parametrized according to the pressure chamber measurements and published data for wheat, extracted from a root hydraulic properties database (Baca Cabrera et al. 2024) (Supplementary Table S1). The age dependency of *k*_r_ and *k*_x_ was modeled using linear piecewise functions, analogously to (Meunier et al. 2018) for maize. Parameterization uncertainty was addressed through a sensitivity analysis, as in Baca Cabrera et al. (2024).

### Statistical analysis

All statistical analyses were conducted in R v.4.4.1 (R Core Team 2024). Kolmogorov–Smirnov tests were applied to compare the diameter distribution of the different root types (crown, seminal, and lateral) across all cultivars. Linear mixed models were performed to test the effect of breeding (expressed as year of cultivar release) on the following traits obtained from root sampling: number of seminal and crown roots, total number of axes (i.e. the sum of seminal and crown roots), average diameters, and branching density. As the field experiment was repeated in 2 consecutive growing seasons, the experimental year was included as the random factor in the models. For the *K*_rs_ measurements, the effect of breeding was tested using linear regression models. Additionally, differences among cultivars (defined as categorical variables) were tested applying ANOVA and Tukey post hoc tests to assess whether the observed effects in the linear models were driven by specific cultivars (e.g. differences between the oldest and newest cultivars). In all cases, we used plant averages for the statistical analyses (*n* = 27–30 for shovelomics traits and *n* = 8 to 12 for *K*_rs_ measurements).

All dependent variables were log-transformed to meet model validation criteria. Models were validated by testing the residuals for normality using the Shapiro–Wilk test (Supplementary Table S3) in combination with visual inspection of Q–Q plots. For plotting purposes, data were back-transformed (Figs. 2 and 3), and the %-change per year (Table 2, Figs. 2 and 3) was calculated for all traits by back-transforming the slope of the linear regression models using the formula: %-change per year = exp (slope—1) × 100. The R packages nlme (Pinheiro et al. 2023) and ggplot2 (Wickham 2016) were used for fitting linear mixed models and data plotting, respectively.

## Supplementary Material

kiaf166_Supplementary_Data

## Data Availability

Data supporting the findings of this study are available within the paper, within its Supplementary data or on request.

## Supplementary data

The following materials are available in the online version of this article.

**Supplementary Figure S1.** The relationship between tiller number and crown root number across cultivars.

**Supplementary Figure S2.** The relationship between year of cultivar release and normalized whole root system conductance.

**Supplementary Figure S3.** The relationship between plant age and simulated root traits in 2 wheat cultivars.

**Supplementary Figure S4.** Transpiration dynamics measured after flowering in the field experiment.

**Supplementary Table S1.**  *P*-values of Tukey post-hoc comparisons of different parameters between cultivars.

**Supplementary Table S2.** List of modified input parameters for the *K*_rs_ simulation with CPlantBox.

**Supplementary Table S3.**  *P*-values of Shapiro–Wilk tests assessing the normality of residuals from linear (mixed) models for all investigated root traits.

## Dive Curated Terms

The following phenotypic, genotypic, and functional terms are of significance to the work described in this paper:

MES CHEBI: CHEBI:39010

crown CHEBI: CHEBI:37409

crown root number Planteome: TO:0002685

root AmiGo: PO:0009005
