# Supplementary material for: Decreased root hydraulic traits in German winter wheat cultivars over 100 years of breeding
Source: Plant Physiol. 2025 Apr 24;198(1):kiaf166. doi: 10.1093/plphys/kiaf166 (PMC12053364; doi:10.1093/plphys/kiaf166)
Supplement: kiaf166_Supplementary_Data [file kiaf166_supplementary_data.pdf]

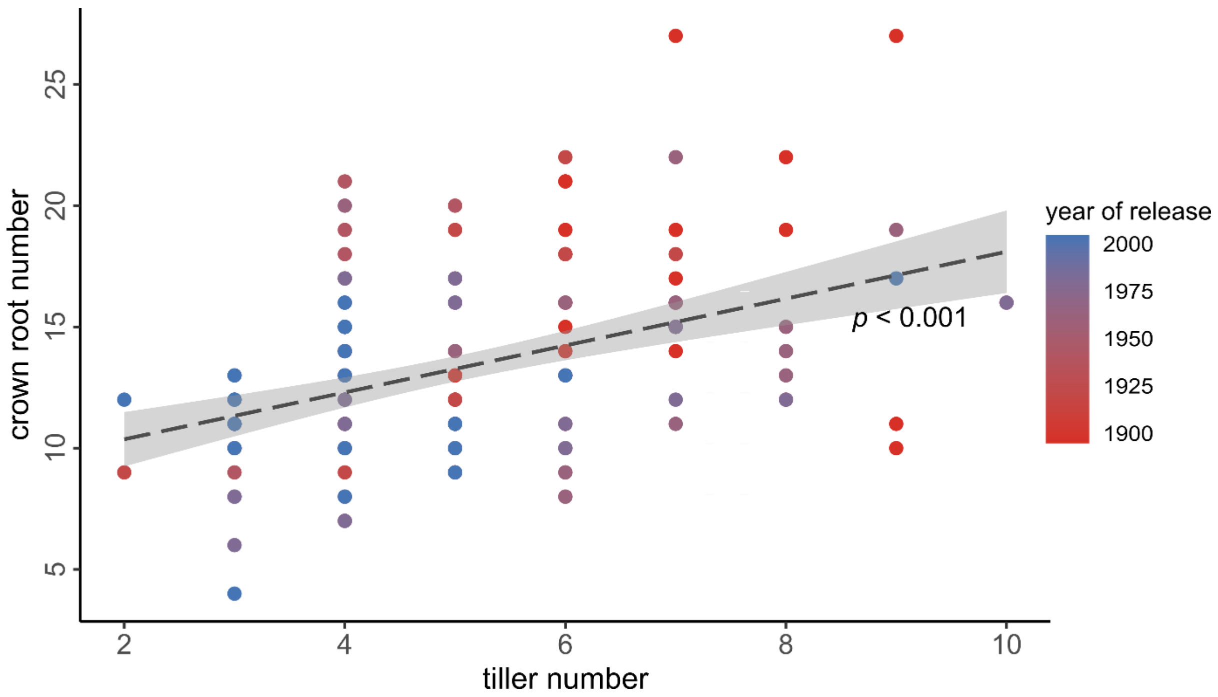

Supplementary Figure S1. The relationship between tiller number and crown root number, across cultivars. The points represent individual wheat plants (*T. aestivum*) grown in the field (n = 27–32 plants). The color scale indicates the year of release of the different cultivars. The dashed line and shaded area represent the ordinary least squares linear regression line ± 95% confidence interval. The p-value for the regression slope (t-test) is shown in the figure.

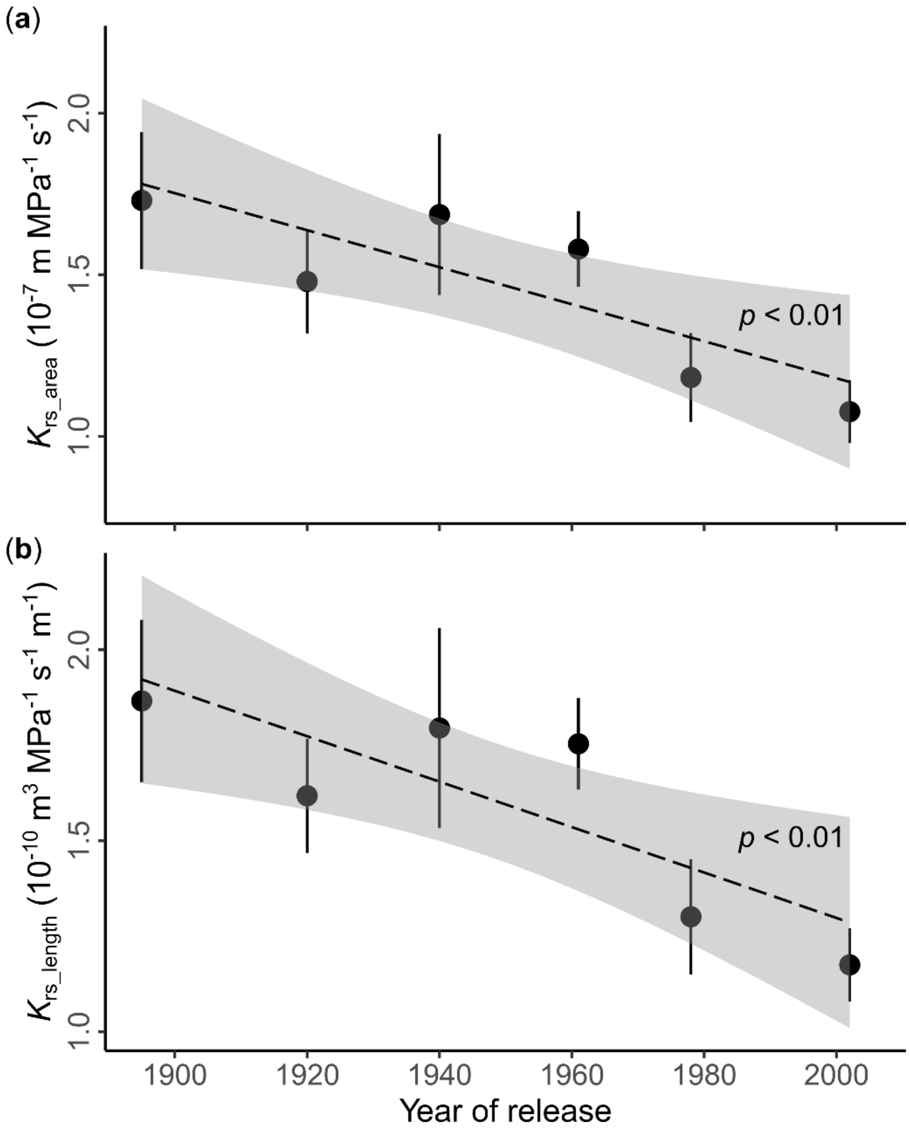

Supplementary Figure S2. The relationship between year of cultivar release and normalized whole root system conductance. Whole root system conductance was normalized by the root system surface area (K_{rs_area}) (**a**) or by the total root length (K_{rs_length}) (**b**). Data points and error bars correspond to pressure chamber measurements in 10–12d old plants and represent the mean ± SE ($n = 8 – 12$ plants). The dashed line and the shaded area represent the ordinary least squares linear regression line ± CI95%. The p-values for the regression slopes (t-test) are shown in the figures.

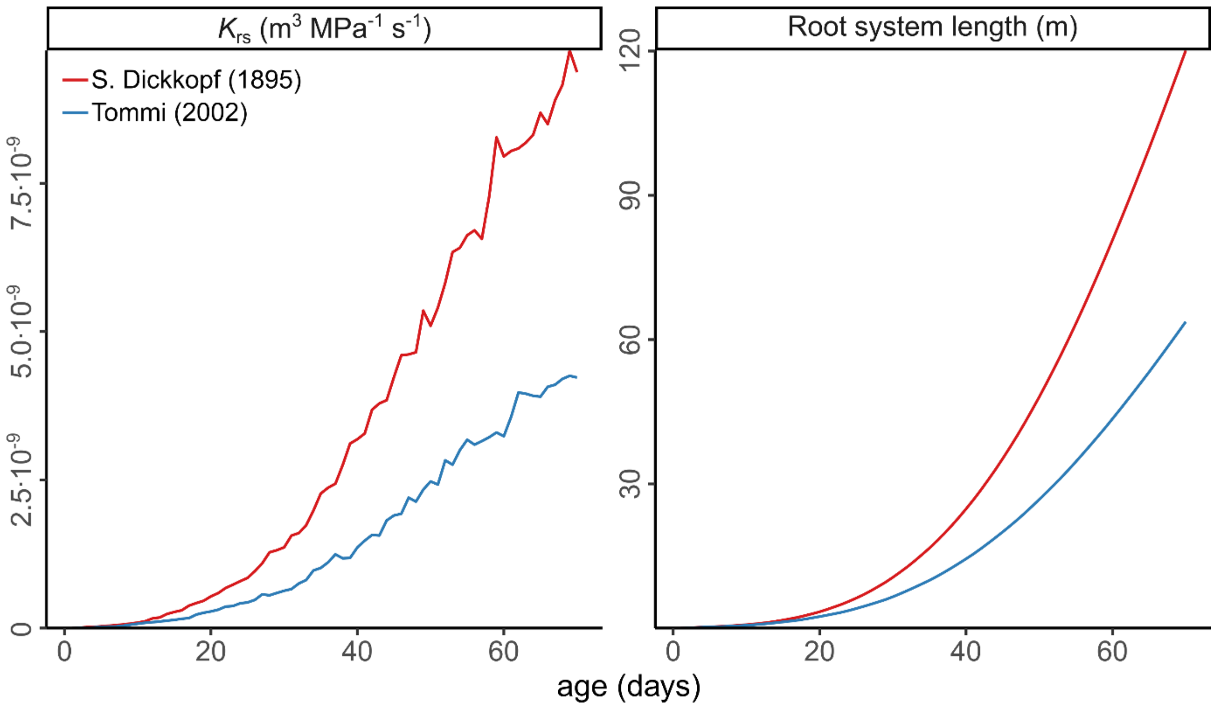

Supplementary Figure S3: The relationship between plant age and simulated root traits in two wheat cultivars. Results correspond to CPlantBox simulations for 70 days, parametrized according to Table S1 for cultivars S.Dickkopf (release year 1895, red lines) and Tommi (release year 2002, blue lines). The left panel shows the whole root system conductance (K_{rs}), and the right panel shows the root system length, based on the average genotype-specific parameterization (n = 1 simulation run).

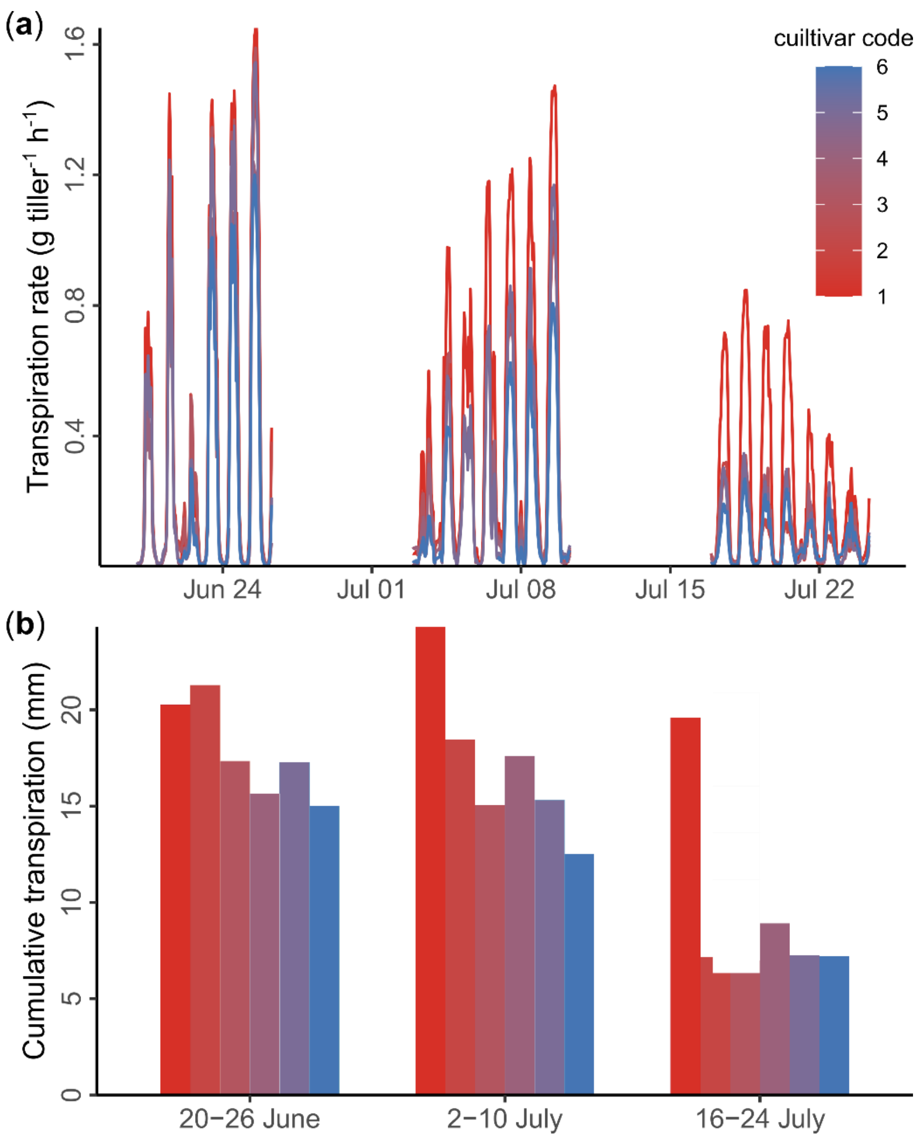

Supplementary Figure S4: Transpiration dynamics measured after flowering in the field experiment. Transpiration rate per tiller (**a**) and cumulative canopy transpiration (**b**) for six different German wheat cultivars grown in the field. Measurements were performed using SGA3-WS (Dynamax Inc., Houston, USA) sap flow sensors. Sensors operate based on the stem heat balance method (Langensiepen et al., 2014). Visualized are the means of five sensors per cultivar, during three different sampling periods after flowering. The cultivars represent over 100 years of breeding history based in their year of release: (1) S. Dickkopf – 1895, (2) SG v. Stocken – 1920, (3) Heines II – 1940, (4) Jubilar – 1961, (5) Okapi – 1978, and (6) Tommi – 2002.

Supplementary Table S1. List of modified input parameters for K_{rs} simulation with CPlantBox. Root morphologic parameters and tiller number were obtained with a slightly modified 'shovelomics' method for wheat (York, 2018), during the tillering phase (BBCH < 30). Hydraulic properties of root segments (radial conductivity k_r and axial conductance k_x) were parametrized based on pressure chamber measurements of the entire root system of young wheat plants (10–12 days, no crown roots) and segment-scale data on wheat and other grasses (Doussan et al., 1998; Bramley et al., 2009; Knipfer and Fricke, 2011; Ahmed et al., 2018), extracted from a root hydraulic properties database (Baca Cabrera et al., 2024). Two parameter sets were defined, representing the oldest (S. Dickkopf, 1895) and the most modern cultivars (Tommi, 2002). The remaining input parameters required in CPlantBox (not shown here) were taken from Giraud et al. (2023, Table S2-S5).

	**Cultivar name (year of release)**	
**Parameter**	S. Dickkopf (1895)	Tommi (2002)
Tiller number	8	5
Seminal root radius (cm)	0.015	0.015
Crown root radius (cm)	0.03	0.03
Lateral root radius (cm)	0.01	0.01
Inter-lateral distance (cm)	0.95	0.95
k_r root tip (m MPa^{-1} s^{-1})	1.5×10^{-7}	1.1×10^{-7}
k_r root base (m MPa^{-1} s^{-1})	1.5×10^{-8}	1.1×10^{-8}
k_x seminal root tip (m^4 MPa^{-1} s^{-1})	8.0×10^{-12}	8.0×10^{-12}
k_x seminal mid-root (m^4 MPa^{-1} s^{-1})	4.0×10^{-11}	4.0×10^{-11}
k_x seminal root base (m^4 MPa^{-1} s^{-1})	6.8×10^{-10}	6.8×10^{-10}
k_x crown root tip (m^4 MPa^{-1} s^{-1})	2.4×10^{-11}	2.4×10^{-11}
k_x crown mid-root (m^4 MPa^{-1} s^{-1})	8.3×10^{-11}	8.3×10^{-11}
k_x crown root base (m^4 MPa^{-1} s^{-1})	1.2×10^{-9}	1.2×10^{-9}
k_x lateral root tip (m^4 MPa^{-1} s^{-1})	8.0×10^{-13}	8.0×10^{-13}
k_x lateral mid-root (m^4 MPa^{-1} s^{-1})	4.0×10^{-12}	4.0×10^{-12}
k_x lateral root base (m^4 MPa^{-1} s^{-1})	6.8×10^{-11}	6.8×10^{-11}

Supplementary Table S2. *p*-values of ANOVA (**a**) and Tukey post-hoc tests (**b**) for crown root diameter and number of root axes. In (**b**) the studied cultivars (CV) are numbered according to their year of release: (1) S. Dickkopf – 1895, (2) SG v. Stocken – 1920, (3) Heines II – 1940, (4) Jubilar – 1961, (5) Okapi – 1978, (6) Tommi – 2002. Significant results (p<0.05) are given in bold type.

	Crown root diameter	Crown root number	Seminal root number	Total axes number
(**a**) ANOVA	**0.02**	**<0.01**	**<0.001**	**<0.001**
(**b**) Tukey post-hoc				
CV1 vs CV2	**0.04**	**<0.05**	**<0.001**	**<0.001**
CV1 vs CV3	0.92	0.19	**<0.001**	**<0.001**
CV1 vs CV4	0.90	0.10	**<0.001**	**<0.001**
CV1 vs CV5	1.0	**<0.05**	**<0.01**	**<0.001**
CV1 vs CV6	0.84	**<0.001**	**<0.001**	**<0.001**
CV2 vs CV3	0.30	0.95	0.67	1.0
CV2 vs CV4	0.37	1.0	0.12	0.98
CV2 vs CV5	**0.02**	1.0	1.0	1.0
CV2 vs CV6	0.49	0.89	0.98	0.82
CV3 vs CV4	1.0	1.0	0.86	0.97
CV3 vs CV5	0.79	0.97	0.46	1.0
CV3 vs CV6	1.0	0.33	0.97	0.74
CV4 vs CV5	0.76	1.0	**<0.05**	0.98
CV4 vs CV6	1.0	0.56	0.43	0.99
CV5 vs CV6	0.68	0.83	0.92	0.78

Supplementary Table S3. *p*-values of Shapiro-Wilk tests assessing the normality of residuals from linear (mixed) models for all investigated root traits. Root traits were log-transformed prior to model fitting. Plant averages were used for statistical analyses (n = 27−30 for shovelomics traits and n = 8–12 for K_{rs} measurements)

**Parameter**	*p*-value	W
crown root diameter (mm)	0.34	0.991
seminal root diameter (mm)	0.37	0.991
lateral root diameter (mm)	0.27	0.990
crown root number	0.42	0.992
seminal root number	0.09	0.986
total axis number	0.29	0.990
tiller number	0.44	0.992
branching density (cm^{-1})	0.08	0.986
root surface area	0.09	0.966
total root length	0.15	0.971
K_{rs}	0.50	0.982
K_{rs_area}	0.26	0.976
K_{rs_length}	0.53	0.982

References

Ahmed MA, Zarebanadkouki M, Meunier F, Javaux M, Kaestner A, Carminati A (2018) Root type matters: measurement of water uptake by seminal, crown, and lateral roots in maize. J Exp Bot **69**: (5)1199–1206

Baca Cabrera JC, Vanderborght J, Couvreur V, Behrend D, Gaiser T, Nguyen TH, Lobet G (2024) Root hydraulic properties: an exploration of their variability across scales. Plant Direct **8**: e582

Bramley H, Turner NC, Turner DW, Tyerman SD (2009) Roles of morphology, anatomy, and aquaporins in determining contrasting hydraulic behavior of roots. Plant Physiol **150**: 348–364

Doussan C, Vercambre G, Pagè L (1998) Modelling of the hydraulic architecture of root systems: an integrated approach to water absorption—distribution of axial and radial conductances in maize. Ann Bot **81**: 225–232

Giraud M, Gall SL, Harings M, Javaux M, Leitner D, Meunier F, Rothfuss Y, van Dusschoten D, Vanderborght J, Vereecken H, et al (2023) CPlantBox: a fully coupled modelling platform for the water and carbon fluxes in the soil–plant–atmosphere continuum. in Silico Plants **5**: diad009

Knipfer T, Fricke W (2011) Water uptake by seminal and adventitious roots in relation to whole-plant water flow in barley (*Hordeum vulgare* L.). J Exp Bot **62**: 717–733

Langensiepen M, Kupisch M, Graf A, Schmidt M, Ewert F (2014). Improving the stem heat balance method for determining sap-flow in wheat. Agric For Meteorol **186**: 34–42.
